# Sample size re-estimation in paired comparative diagnostic accuracy studies with a binary response

**DOI:** 10.1186/s12874-017-0386-5

**Published:** 2017-07-14

**Authors:** Gareth P. J. McCray, Andrew C. Titman, Paula Ghaneh, Gillian A. Lancaster

**Affiliations:** 1 Institute of Primary Care and Health Sciences, Keele University, David Weatherall Building, Stoke-on-Trent, ST5 5BG UK; 2 Department of Mathematics and Statistics, Lancaster University, Fylde College, Lancaster, LA14YF UK; 3 Institute of Translational Medicine, University of Liverpool, Cedar House, L69 3GE, Ashton St, Liverpool, L3 5PS UK

**Keywords:** Interim analysis, Sample-size re-estimation, Study design, Diagnostic accuracy, Sensitivity, Specificity

## Abstract

**Background:**

The sample size required to power a study to a nominal level in a paired comparative diagnostic accuracy study, i.e. studies in which the diagnostic accuracy of two testing procedures is compared relative to a gold standard, depends on the conditional dependence between the two tests - the lower the dependence the greater the sample size required. A priori, we usually do not know the dependence between the two tests and thus cannot determine the exact sample size required. One option is to use the implied sample size for the maximal negative dependence, giving the largest possible sample size. However, this is potentially wasteful of resources and unnecessarily burdensome on study participants as the study is likely to be overpowered. A more accurate estimate of the sample size can be determined at a planned interim analysis point where the sample size is re-estimated.

**Methods:**

This paper discusses a sample size estimation and re-estimation method based on the maximum likelihood estimates, under an implied multinomial model, of the observed values of conditional dependence between the two tests and, if required, prevalence, at a planned interim. The method is illustrated by comparing the accuracy of two procedures for the detection of pancreatic cancer, one procedure using the standard battery of tests, and the other using the standard battery with the addition of a PET/CT scan all relative to the gold standard of a cell biopsy. Simulation of the proposed method illustrates its robustness under various conditions.

**Results:**

The results show that the type I error rate of the overall experiment is stable using our suggested method and that the type II error rate is close to or above nominal. Furthermore, the instances in which the type II error rate is above nominal are in the situations where the lowest sample size is required, meaning a lower impact on the actual number of participants recruited.

**Conclusion:**

We recommend multinomial model maximum likelihood estimation of the conditional dependence between paired diagnostic accuracy tests at an interim to reduce the number of participants required to power the study to at least the nominal level.

**Trial registration:**

ISRCTN ISRCTN73852054. Registered 9th of January 2015. Retrospectively registered.

## Background

An assessment of diagnostic accuracy is crucial in the development of medical testing procedures [1]. Comparing the accuracy of these procedures in terms of their sensitivities and specificities [2, 3] relative to a gold standard, is essential to ensuring that the most appropriate tests are deployed in the clinical setting [4, 5]. The focus of this paper is sample size re-estimation in the comparison of two candidate tests to a gold standard. However, diagnostic accuracy studies do not necessarily involve comparisons; many such studies report the accuracy of a single test.

At the outset of a study, a sample size is calculated based on assumptions made about the expected changes in sensitivity and specificity and, in a prospective design, the likely prevalence of the condition to be tested for in the sample. However, the initial assumptions about parameters in the study, especially the conditional dependence between the two tests, may be revealed to be inaccurate, resulting in a potentially over- or under-powered study. A planned interim analysis can allow the study’s sample size to be updated based on the data already collected. This involves utilising the information observed at the interim stage to refine the sample size estimate. A resulting increase in sample size allows the time, cost and patient discomfort already invested in the study to yield valid results while a decrease in sample size means that less time and cost will be expended overall and patients will not needlessly undergo unnecessary testing [6].

There are well-established methodologies for interim sample size re-estimation in treatment studies for continuous and normally distributed response variables [7–11], some of which provide mechanisms to maintain blinding in the study [8–10]. Methods also exist for the re-estimation with binary response variables [12, 13], and mechanisms to maintain blinding have been proposed in this more complex situation where the variance and mean parameters are not separable [14]. Proschan [15] gives an overview of sample size re-estimation procedures based on a nuisance parameter. Specifically, procedures for determining the difference of means between two samples with a common, unknown, variance and difference in proportions between two groups, with an unknown overall proportion, are considered. In the case of normally distributed data, the independence of the sample variance and sample mean ensures that the validity of estimates is unaffected by the interim sample size re-estimation and this is shown to hold asymptotically in the binary case. However, Proschan does not consider the case of paired data which is the focus of the current paper. Furthermore, the implications of sample size re-estimation in the context of comparative diagnostics studies, inherently different from those in treatment (randomised controlled) studies [16], have not been fully explored in the statistical literature.

A number of salient differences in interim analysis between studies comparing diagnostic tests and those comparing treatments are highlighted in Gerke et al*.* [5] and Gerke et al*.* [16]. Firstly, in paired diagnostics accuracy studies, full blinding is often not possible, specifically, certain types of test may not be able to be blinded from the patient, the person administering the test, the person interpreting the test, or the person measuring the outcome. However, as long as the results of the two-tests which are being compared are temporarily blinded from the person measuring the outcome, this is not a major threat to a study’s validity [17]. In fact, it has the advantage that the patients can benefit from their clinicians knowing the results of both diagnostic tests after testing has taken place. Secondly, in diagnostic accuracy studies, early cessation of the study due to futility is not as easy to establish as in treatment studies. The reasons for this are 1) the fact that treatment studies often test a single outcome while diagnostic studies test two outcomes, sensitivity and specificity, and futility must be established for both simultaneously, and 2) patient outcomes may only be seen further downstream from the test results [18]. Thirdly, the sample size required for a hypothesis test in diagnostic studies, powered to a given level, is closely related to the conditional dependence between the two testing procedures which has been shown to present problems in a number of contexts [5, 19–24]. More specifically, the lower the conditional dependence between the tests, the greater the sample size will be, with the largest sample size being implied by the maximum negative dependence, given the specified alternative hypotheses. This level of conditional dependence between the tests is one of the primary factors driving the required sample size estimate and it is often difficult to estimate a priori. Gerke et al*.* [5] assert that for comparative diagnostic studies, as long as an interim sample size re-estimation is planned it bears no threat to the validity of the study. However, Gerke et al*.* [5] do not provide justification for this assertion and, furthermore, their assertion does not take the inherent uncertainty of the interim data into account. This study aims to present a method and give practical guidelines for its application, for the initial estimation and interim re-estimation of sample size in a paired diagnostic study which will allow utilisation of information on the conditional dependence between tests at the interim to potentially reduce the required sample size while maintaining the approximate nominal statistical power of the experiment as a whole. While we present a method of estimating the size of the conditional dependence to reduce sample size, it should also be noted that there is a body of literature dealing with the problems caused by conditional dependence in other areas [25–27].

The remainder of the article is organised as follows. The methods section outlines sample size estimation methods for paired diagnostic test studies, introduces a motivating example application, and then goes on to propose a new method for re-estimation based on a multinomial likelihood. The results section first provides extensive simulations of the method under various real world conditions and then moves back to apply the sample size re-estimation method proposed in this paper to the motivating example. The article then continues with a brief discussion of the place of this study in the literature and the optimal interim sample size to choose. Finally, the conclusion, summarises and restates the major outcomes of this study.

## Methods

A representation of data from a paired comparative diagnostic accuracy study is given in Table 1. The subjects are initially divided according to whether they are discovered, via the gold standard test, to be diseased or non-diseased. They are then further subdivided as to whether they test positive or negative on tests A and B. For example, the cell *n*_*A*_ represents subjects that were found to have the disease via the gold standard test and also tested positive on both test A and B, while cell *n*_*F*_ denotes subjects who tested negative on the gold standard and test B but positive on test A.

**Table 1:** Paired study design

Diseased				Non-diseased			
		Test B				Test B	
		+ive	-ive			+ive	-ive
Test A	+ive	*n*_*A*_	*n*_*B*_	Test A	+ive	*n*_*E*_	*n*_*F*_
	-ive	*n*_*C*_	*n*_*D*_		-ive	*n*_*G*_	*n*_*H*_

A possible initial sample size calculation, using a normal approximation of the logarithm of the ratio of sensitivities and specificities, and assuming a comparison between a new test, test A, and an existing test, test B, follows from Alonzo et al*.* [21] and a full derivation can be found therein. The experiment, as a whole tests jointly both sensitivity and specificity improvement to pre-specified levels, the sample size is calculated for each and the largest sample size is chosen to power the study. Note that this paper concentrates on the situation in which superiority is tested for both sensitivity and specificity. However, the method elaborated below should be extendable to situations where we are interested in testing non-inferiority in either or both of sensitivity and specificity. For details on the construction of the confidence intervals and hypothesis tests in these situations see *Alonzo* et al*.* [21]. In the case of the estimation of a sample size for superiority, the initial sample size calculation for sensitivity is given by:

1 $$ {n}_{p1}={\left(\frac{Z^{\left(1-\beta \right)}+{Z}^{\left(1-\alpha /2\right)}}{\mathit{\log}{\gamma}_1}\right)}^2\left(\frac{\left({\gamma}_1+1\right){TPR}_B-2 TPPR}{\gamma_1{TPR}_B^2}\right)/\pi $$

where, *α* is the type I error rate of the study and *β* is the power of the study. The main quantity of interest, *γ*_1_, is the ratio of true positive rates=*TPR*_*A*_/*TPR*_*B*_, *TPR*_*B*_ is the true positive rate (sensitivity) on test B, i.e. *TPR*_*B*_ = (*n*_*A*_ + *n*_*C*_) / (*n*_*A*_ + *n*_*B*_ + *n*_*C*_ +  *n*_*D*_), *TPR*_*A*_ is the true positive rate (sensitivity) on test A, i.e. *TPR*_*A*_= (*n*_*A*_ + *n*_*B*_) / (*n*_*A*_ + *n*_*B*_ + *n*_*C*_ +  *n*_*D*_), *TPPR* is the proportion of diseased patients who test positive on both tests, i.e. *TPPR* = *n*_*A*_/( *n*_*A*_ + *n*_*B*_ + *n*_*C*_ +  *n*_*D*_) and *π* is the prevalence of disease. The null hypothesis is that *γ*_1_ = 1, the alternative hypothesis is that *γ*_1_≠1.

For testing superiority of specificity we are interested in the true negative rates so the formula is instead:

2 $$ {n}_{n1}={\left(\frac{Z^{\left(1-\beta \right)}+{Z}^{\left(1-\alpha /2\right)}}{\mathit{\log}{\gamma}_2}\right)}^2\left(\frac{\left({\gamma}_2+1\right){TNR}_B-2 TNNR}{\gamma_2{TNR}_B^2}\right)/\left(1-\pi \right) $$

where, *γ*_2_, the main quantity of interest is the ratio of true negative rates =*TNR*_*A*_/*TNR*_*B*_, *TNR*_*A*_ is the true negative rate (specificity) on test A = (*n*_*G*_ + *n*_*H*_) / (*n*_*E*_ + *n*_*F*_ + *n*_*G*_ +  *n*_*H*_), *TNR*_*B*_ is the true negative rate (specificity) on test B = (*n*_*F*_ + *n*_*H*_) / (*n*_*E*_ + *n*_*F*_ + *n*_*G*_ +  *n*_*H*_), and *TNNR* is the proportion of non-diseased patients who test negative on both tests = *n*_*H*_/( *n*_*E*_ + *n*_*F*_ + *n*_*G*_ +  *n*_*H*_).

It is interesting to note that, following the notation of Vacek [25] and considering the population 2 × 2 table (in Table 1), the conditional dependence of the two tests can be denoted by *e*_*b*_ and *e*_*a.*_, the conditional covariance when the gold standard disease status is positive or negative, respectively [25]. Therefore, the probability of both tests being positive can be expressed as *TPPR* = *TPR*_*A*_ ∙ *TPR*_*B*_ + *e*_*b*_ and the probability of both tests being negative *TNNR* = (1 − *TNR*_*A*_) ∙ (1 − *TNR*_*B*_) + *e*_*a*_. When *e*_*a*_ and *e*_*b*_ = 0 the tests are conditionally independent, when *e*_*a*_ and/or *e*_*b*_ ≠ 0 the response on one test changes the probability of that response on the other test. For example, when *e*_*b*_ > 0 an individual who responds positively on test A is more likely to respond positively on test B.

For initial estimates of *TPPR* and *TNNR*, from Alonzo et al*.* [21] we can use the fact that *TPPR* ≥ (1 + *γ*_1_)*TPR*_*B*_ − 1 and *TNNR* ≥ (1 + *γ*_2_)*TNR*_*B*_ − 1 to estimate the lower bounds of the possible values of *TPPR* and *TNNR*, under the specified hypotheses. The required sample size is largest when *TPPR* = (1 + *γ*_1_)*TPR*_*B*_ − 1 and *TNNR* = (1 + *γ*_2_)*TNR*_*B*_ − 1, thus, these estimates represent the “worst case scenarios” of maximal negative conditional dependence between the tests, conditional on the fixed values of *TPR*_*A*_ and *TPR*_*B*_. The sample size implied by using these levels of *TPPR* and *TNNR* would very likely overpower the study, i.e. more participants will be recruited than is strictly necessary to achieve the power specified by *β*. The required sample size is smallest when the conditional dependence between tests A and B are maximal, conditional on the fixed values of *TPR*_*A*_ and *TPR*_*B*_, i.e. when *TPPR* = *TPR*_*B*_ and *TNNR* = *TNR*_*B*_. The implied sample size in this case would likely underpower the study, i.e. too few participants recruited to reach the power specified by *β*. The sample size in this “best case scenario” can be substantially lower than that in the worst case scenario. Conservatively, it might be thought a good idea to always use the “worst case scenario” implied sample size estimate which will always power the study sufficiently. However, in cases where the recruitment and testing of participants comes at a premium, both financially and in terms of discomfort to the patients, it might be preferable to apply a more nuanced strategy. Furthermore, the sample size implied by the “worst case scenario” implies the highly unlikely condition of a maximal negative conditional dependence between two tests, which are performed on the same patients to detect the same disease. The implied sample size based on this condition is not recommended [28]. One possibility, to enable a more accurate evaluation of the conditional dependence between the two tests, and thus the required sample size, is to perform a planned interim sample size re-estimation using this information to refine the sample size estimate.

At a planned interim, where a proportion of the overall sample size has been collected, we would have some information about the true values of *TPPR*, *TNNR*, *π*, *TPR*_*B*_ and *TNR*_*B*_, however, these values would only come from a limited sample size. The crucial parameters to use in re-estimation are those related to the conditional dependence between the tests, i.e., *TPPR* and *TNNR*, as these values are difficult to estimate and, for these parameters, it is unlikely that research exists which can provide an approximate value. Conversely, the values of, *TPR*_*B*_ and *TNR*_*B*_, the sensitivities and specificities of an established test, may have known values in the literature and these should preferably be used over those from the relatively small interim sample. For the value of *π*,the prevalence, a judgement must be made as to whether the researcher feels that any pre-existing estimate of prevalence would be a more accurate reflection of the true prevalence in the specific study population than any interim estimate. In the example given below, we use values for *TPPR*, *TNNR* and *π* at the interim in the sample size calculation.

Naively, it might appear that interim sample size re-estimation would entail a straightforward replication of eqs. (1) and (2) with *π*, and in the case of (1), *TPPR* or in the case of (2), *TNNR*, replaced with the estimates at the interim point. However, this approach does not effectively take into account the inherent uncertainty in the interim parameter estimates of *TPPR*, *TNNR* and *π*, nor the fact that only a specific range of values for *TPPR* and *TNNR* are actually possible under the alternative hypothesis. An approach which does take these factors into account is re-estimation of the sample size based on maximum likelihood estimation, at the interim, of the parameters in question under a multinomial model. This model is constrained by the hypothesised values of *TPR*_*A*_ , *TPR*_*B*_, *TNR*_*A*_, and *TNR*_*B*_, i.e. the marginals in Table 1.

### Application

The numerical example we use involves an interim sample size recalculation of a study comparing the incremental benefits to sensitivity and specificity of augmenting current methods for diagnosing pancreatic cancer with Positron Emission Tomography (PET) and computed tomography (CT) technologies. The alternative hypotheses were that sensitivity would rise from 81% to 90%, and specificity would rise from 66% to 80%, additionally, the expected prevalence of pancreatic cancer from the literature was 47%.

To calculate the sample size for sensitivity equation 1 was used, taking $\alpha =0.05,\kern0.5em \beta =0.2,\kern0.5em {\widehat{\gamma}}_1=\frac{0.9}{0.81},$ $\widehat{TPR_B}=0.81,$ $\widehat{TPPR}=0.71$, and $\widehat{\pi}=0.47$ gives a sample size of **598**. To calculate the sample size for specificity equation 2 was used taking $\alpha =0.05,\kern0.5em \beta =0.2,{\ \widehat{\gamma}}_2=\frac{0.8}{0.66},$ $\widehat{\ {TNR}_B}=0.66,$ $\widehat{TNNR}=0.46$, and $\widehat{\pi}=0.47$ gives a sample size of **409**. The minimum sample sizes for sensitivity and specificity, given $\widehat{TPPR}=0.81$ and $\widehat{TNNR}=0.66$, are **186** and **106**, respectively. Given the disparity between the minimum and maximum sample size estimates it was decided to re-assess the sample size at a planned interim.

Table 2 gives the results after data from 187 participants had been collected. The observed values at the interim are: $\widehat{TPPR}=0.80$, $\widehat{TNNR}=0.66$ and $\widehat{\pi}=0.44$. Taking a naive approach and plugging these values directly into equations 1 and 2 the implied sample sizes for sensitivity become **242** and for specificity **100**, giving a total sample size for the study of **242** (or **342** and **145,** respectively, had we also used the interim values of *TPR*_*B*_ and *TNR*_*B*_). However, this method does not take into account the fact that $\widehat{TPPR}$ and$\widehat{\  TNNR}$ are random variables and we are actually interested in the true value of the probability of *TPPR* and *TNNR* under the specified alternative hypothesis. In fact, had the observed value for *TPPR* been equal to 0.86, the sample size given via the naive method would have been **−22**, given the fact that $\widehat{TPPR}$ would have been larger than both *TPR*_*A*_ and *TPR*_*B*_. Clearly, the naive method, which uses the random value of a single cell, is inappropriate and a method that uses information about the value of *TPPR* from all of the observed cells and the specified marginals is required.

**Table 2:** Interim PET diagnostic study results

Diseased patients				Non-diseased patients			
		Pre-PET				Pre-PET	
		+ive	-ive			+ive	-ive
Post-PET	+ive	66	3	Post-PET	+ive	21	4
	-ive	3	10		-ive	11	69

### Sample size re-estimation via maximum likelihood estimation of *TPPR*

For illustration purposes, we will discuss the re-estimation of the sample size for sensitivity, the estimation procedure for specificity is analogous. Taking *TPR*_*A*_ as the test with the highest expected diagnostic utility, i.e. the “new” test whose performance we are comparing to the “standard”, the probabilities corresponding to the cells in Table 1, given the situation of the maximally negative conditional dependence between the tests are: *p*_1_ = *TPR*_*B*_ − (1 − *TPR*_*A*_), *p*_2_ = 1 − *TPR*_*B*_, *p*_3_ = 1 − *TPR*_*A*_, *p*_4_ = 0. The probabilities of the cells when the conditional dependence between *TPR*_*A*_ and *TPR*_*B*_ is at its maximally positive are given by: *p*_1_ = *TPR*_*B*_, *p*_2_ = *TPR*_*A*_ − *TPR*_*B*_, *p*_3_ = 0, *p*_4_ = 1 − *TPR*_*A*_. We could alternatively specify these cell probabilities according to the covariance between the two tests. Specifically, Vacek [25] gives the maximum value of the covariance as *TPR*_*B*_ (1 − *TPR*_*A*_) and the minimum value as −(1 − *TPR*_*A*_)(1 − *TPR*_*B*_). Thus, the maximum and minimum values for the cells can be ascertained by finding the product of the marginal probabilities associated with a cell and adding the minimum or maximum value of covariance, for cells *p*_1_ and *p*_4_, or subtracting the values of covariance for cells *p*_2_ and *p*_3_. For example, the minimum value for *p*_1_ = *TPR*_*A*_ ∙ *TPR*_*B*_ − (1 − *TPR*_*A*_)(1 − *TPR*_*B*_). Between the minimum and maximum values lies every permissible joint configuration. Let these possible joint configurations be expressed as vector, **p**, with *p*_1_ = *TPPR*,where ${\sum}_{i=1}^4{\mathbf{p}}_i=1,{\  p}_1+{p}_2 = {TPR}_A$ and *p*_1_ + *p*_3_ = *TPR*_*B*_.

When the conditional dependence is maximally positive the sample size required is the smallest, when it is maximally negative the sample size required is at its largest. At the beginning of the experiment we do not know which of these possible levels of conditional dependence our data were generated under and thus we use the, usually overly conservative, largest possible sample size estimate.

However, at the interim we can use our observed data to infer a likelihood of that data having been generated under each of the permissible joint configurations of cell probabilities given the implied range of probabilities under a multinomial model. A simple method of extracting an estimate of TPPR is to maximise the likelihood function of the interim data given the values of **p** implied by the marginal probabilities:

3 $$ \mathcal{L}\left(\boldsymbol{p}| x\right)=\kern0.5em \prod_{i=1}^4{\boldsymbol{p}}_i^{x_i} $$

where **p** is the vector of joint probabilities defined above and *x* are the observed cell frequencies. The constraints imposed on the above multinomial likelihood make the parameter space one dimensional, thus, substituting the constraints in order to express the likelihood in terms of *p*_1_, gives:

4 $$ \mathcal{L}\left({p}_1| x\right)={p}_1^{x_1}{\left(\ {TPR}_A - {p}_1\right)}^{x_2}{\left(\ {TPR}_B - {p}_1\right)}^{x_3}{\left(1 - {TPR}_A - {TPR}_B + {p}_1\right)}^{x_4} $$

$$ {\  p}_1\in \left[\ {TPR}_B-\left(1 - {TPR}_A\right),{\  TPR}_B\right] $$

Code to estimate this in R, via optimisation of the negative log-likelihood, is in the Appendix. In effect, this method bounds the value for the conditional dependence between the minimum and maximum values under the specified marginals and then uses information from the frequency values of the four cells of the table to infer the most probable value of *p*_1_. We can use this estimate of${\ \widehat{p}}_1$ as our value of $\widehat{TPPR}$ and use the observed value of the prevalence (if required) as our measure of $\widehat{\pi}$ in equation 1 to re-estimate the sample size at the interim.

## Results

### Simulation studies

In order to verify the integrity of the method for sample size re-estimation described and applied above a series of simulation studies were carried out. The objectives of these studies were to assess the implications of re-estimating a sample size based on data already collected on the type I and II error rates under various permutations of parameters. The type II error rate should be as close to nominal as possible (i.e. 0.8 in the example above), and the type I error rate should be minimally affected by the re-estimation.

**Fig. 1:**
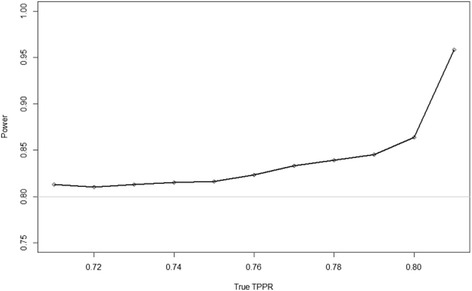
Simulated power of sample size specified by the true TPPR in equation 1 when TPR_A_=0.9, TPR_B_=0.81 and π=0.45

It should be noted that the statistical power provided by the sample size implied by the Alonzo et al. [21] method (when no re-estimation is undertaken) is related to the level of conditional dependence between the tests, Fig. 1 illustrates this relationship. In total 100,000 replications were generated under the specified true alternative hypothesis (i.e. *γ*_1_ = 0.9/0.81 = 1.11), for the example situation above, at various levels of conditional dependence between the two tests. The number of replications 100,000, is more than required, however as the computing time to calculate these was trivial, there was little cost in simulating to this level of accuracy. This number of simulations was used throughout this paper. In all cases in Fig. 1 the simulated power was higher than nominal but where the conditional dependence was highest the power was greatly over specified. As the conditional dependence tends towards becoming maximally positive, i.e. as TPPR tends towards its maximal value, the cell *n*_*C*_ tends towards 0. This means that the asymptotic assumptions underlying formulae 1 and 2 and those underlying the significance test no longer hold. However, this should not be of too great a concern, with regards to balancing the minimisation of the required sample size estimate with the statistical power of the experiment, as the instances where the power is over specified are when the sample size is lowest. Additional conservatism at positive levels of conditional dependence has a significantly lesser impact on the overall sample size than it would have at the end of the continuum where the conditional dependence is negative. Whatever the case may be, it should be noted that the results of re-estimation will follow a similar pattern.

**Fig. 2:**
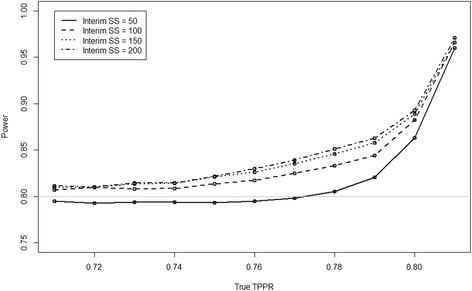
Simulated power of re-estimation method across various interim sample sizes and levels of true TPPR when TPR_A_ = 0.9, TPR_B_ = 0.81 and π = 0.45

In the first set of simulations, which aim to assess the stability of the type II error rate, data are generated under the conditions *TPR*_*A*_ = 0.9, *TPR*_*B*_ = 0.81, *π* = 0.45, while the sample sizes at the interim are varied between 50 and 200 and the values for *TPPR* are varied between 0.71 and 0.81. The null hypothesis is: *TPR*_*A*_/*TPR*_*B*_ = 1, and our data were simulated under the alternative hypothesis *TPR*_*A*_ = 0.9 and *TPR*_*A*_ = 0.81, with varying levels of conditional dependence within the implied limits. Figure 2 shows how the power of the experiment overall (i.e. using the data from both before and after sample size re-estimation) varies as a function of the interim sample size and the true value of TPPR. As expected the values follow the same pattern as that in Fig. 1. The minimum of the nominal power, or very close to it, was achieved at all levels of conditional dependence and at all interim sample sizes.

Table 3 provides information about the mean sample size, bias, coverage and Root Mean Squared Error (RMSE) (from the value specified by equation 1 using the true value of *TPPR* for the simulated data) under the combinations of conditional dependences and interim sample size. The sample sizes implied by Equation 1 for maximal and minimal levels of conditional dependence are **194** and **625**, respectively. The interim sample sizes of 50, 100, 150 and 200 were chosen to illustrate the effects of choosing various interim sample sizes that were smaller than the total sample size of 242 calculated by the Alonzo method described above for our application.

**Table 3:** Mean sample size (S.D.), bias, coverage and RMSE of simulated sample sizes with varying interim sample size estimates and true levels of TPPR when TPR_A_= 0.9, TPR_B_ = 0.81 and Prevalence = 0.45. (N = interim sample size)

	Mean sample size				Bias			
	*N* = 50	*N* = 100	*N* = 150	*N* = 200	*N* = 50	*N* = 100	*N* = 150	*N* = 200
TPPR								
0.81	217(77)	202(35)	198(23)	205(17)	−0.00091	−0.00027	0.00018	0.00048
0.80	256(114)	241(71)	238(56)	241(48)	−0.00031	0.00035	0.00062	0.00064
0.79	297(139)	283(92)	281(72)	282(62)	−0.00007	0.00069	0.00072	0.00068
0.78	338(155)	326(105)	325(83)	325(70)	0.00045	0.00056	0.00082	0.00062
0.77	381(166)	371(114)	369(89)	369(75)	0.00043	0.00054	0.00058	0.00050
0.76	423(170)	415(118)	413(92)	413(78)	0.00054	0.00035	0.00054	0.00041
0.75	465(171)	460(118)	457(93)	456(79)	0.00069	0.00056	0.00029	0.00033
0.74	506(166)	503(115)	501(91)	500(78)	0.00029	0.00028	0.00031	0.00031
0.73	546(156)	546(107)	545(86)	543(73)	0.00047	0.00045	0.00022	0.00022
0.72	585(143)	588(95)	88(76)	586(65)	0.00043	0.00027	0.00017	0.00022
0.71	621(124)	629(75)	630(59)	629(50)	0.00024	0.00037	0.00033	0.00019
	Coverage				RMSE			
	*N* = 50	*N* = 100	*N* = 150	*N* = 200	*N* = 50	*N* = 100	*N* = 150	*N* = 200
TPPR								
0.81	0.923	0.925	0.924	0.923	80	36	23	18
0.8	0.936	0.937	0.936	0.936	115	71	62	48
0.79	0.942	0.943	0.944	0.943	140	92	72	62
0.78	0.947	0.947	0.947	0.946	156	105	80	70
0.77	0.948	0.948	0.949	0.947	166	114	89	75
0.76	0.949	0.950	0.950	0.949	171	118	92	78
0.75	0.950	0.950	0.949	0.950	171	119	93	79
0.74	0.950	0.950	0.950	0.950	166	115	91	78
0.73	0.950	0.951	0.951	0.951	156	107	86	73
0.72	0.951	0.949	0.950	0.951	143	95	76	65
0.71	0.949	0.949	0.950	0.950	124	75	59	50

An increasing interim sample size does not have that great an impact on the average estimated sample size. However, it does have a large impact on the RMSE. Thus, choosing a larger interim sample size at which to re-estimate will ensure a more accurate sample size re-estimate in individual cases, meaning that the experiment will be more likely to be powered to the appropriate level while recruiting as few participants as possible. Of course, if the interim sample size is chosen to be too large then there is a risk of having already recruited too many participants at the interim. Therefore, some sensible trade-off is required. The bias and coverage seem to be at acceptable levels although the coverage does dip when the conditional dependence between the tests is high.

A second set of simulations was run to assess the performance of the method under the null hypothesis where ${\gamma}_1=\frac{{\  TPR}_A}{{\  TPR}_B}=1$. Table 4 shows the cell probabilities for these simulations. Rather than report across the entire range only the minimum, 50% (i.e., median) and maximum levels of *TPPR* are reported.

**Table 4:** Simulation settings to estimate Type I error

*p*_*A*_	*p*_*B*_	*p*_*C*_	*p*_*D*_	*TPR*_*A*_	*TPR*_*B*_	*γ*
0.81	0.045	0.045	0.10	0.855	0.855	1
0.76	0.095	0.095	0.05	0.855	0.855	1
0.71	0.145	0.145	0.00	0.855	0.855	1

Table 5 shows the type I error rate, mean sample size, bias, coverage and RMSE of simulated sample sizes under various simulation settings. At all levels of conditional dependence and at all interim sample sizes the type I error rate is close to the specified levels. Again, the inference to be made from the RMSE value is that a larger sample size provides a more accurate estimate of the full sample size required, reducing the extent to which an experiment will be over or underpowered in individual cases. The bias and coverage also appear to be at acceptable levels.

**Table 5:** Type I error rate, Mean sample size (S.D.), bias, coverage and RMSE of simulated sample sizes under various simulation settings

	*N* = 50	*N* = 100	*N* = 150	*N* = 200
	Type I error rate			
TPPR				
0.81	0.050	0.050	0.050	0.050
0.76	0.050	0.050	0.050	0.050
0.71	0.050	0.050	0.050	0.050
	Mean sample size			
TPPR				
0.81	304(121)	298(78)	297(61)	296(52)
0.76	463(159)	457(107)	454(84)	453(71)
0.71	627(118)	631(74)	630(58)	629(50)
	Bias			
TPPR				
0.81	0.00207	0.00224	0.00198	0.00186
0.76	0.00147	0.00110	0.00100	0.00087
0.71	0.00021	0.00023	0.00005	−0.00019
	Coverage			
TPPR				
0.81	0.952	0.951	0.950	0.951
0.76	0.950	0.949	0.949	0.949
0.71	0.948	0.949	0.950	0.949
	RMSE			
TPPR				
0.81	164	130	119	115
0.76	168	117	95	83
0.71	118	74	59	50

Table 6 gives the results of a range of simulations undertaken at various values of *TPR*_*A*_, *TPR*_*B*_ and *π* in both true alternative and null cases. Regarding the best sample size to specify at the interim, a possible balance to be struck between a suitably large interim sample, which would increase the precision of the measure of conditional dependence, and minimising the overall experimental sample size would be to take the minimal possible sample size for the experiment as a whole at the interim. In this way, the interim sample could never be larger than the overall required sample size, which means that it is impossible to collect more data than actually needed. Yet, the minimum possible overall sample size represents a significant proportion of the total experimental sample size. Thus, for the values in Table 6, the sample size re-estimate was conducted at the number implied by equation 2, when *TPPR* is at maximal value given the marginals. The maximum positive, mid-range and maximum negative levels of *TPPR* were reported to show a range of values across different levels of *TPPR*. The mean sample size is provided in parentheses in order to allow intuition about the reduction in the sample size this method brings. In all cases, where data were generated under the true alternative hypothesis, the simulated power is above or very close to the nominal value. Furthermore, in all cases where data were generated under the true null hypothesis the size is close to the nominal value. Comparing the mean sample sizes given for the maximal and mid-point *TPPR*s against the fixed values that would be used if Alonzo et al. [21] had been followed we can see that the sample size re-estimation method outlined above can dramatically reduce the required sample size to power an experiment to the minimum of a nominal level.

**Table 6:** Simulated type I and II error rates and fixed maximal sample size values under various true values of TPR_A_, TPR_B_ and prevalence across various levels of conditional dependence (average sample size given in brackets)

	TPRb	TPRa	prev = 0.1			prev = 0.3			prev = 0.5		
			Alternative	Null	Fixed	Alternative	Null	Fixed	Alternative	Null	Fixed
Maximum positive TPPR	0.5	0.6	0.979(871)	0.049(1255)	7084	0.977(289)	0.05(417)	2361	0.977(172)	0.048(249)	1417
	0.5	0.7	0.98(434)	0.047(616)	1585	0.98(143)	0.047(204)	528	0.978(85)	0.049(122)	317
	0.5	0.8	0.986(297)	0.048(401)	622	0.985(98)	0.047(133)	207	0.985(58)	0.048(79)	124
	0.5	0.9	0.991(232)	0.048(279)	303	0.99(76)	0.046(92)	101	0.989(45)	0.046(54)	61
	0.6	0.7	0.976(858)	0.047(1244)	5505	0.975(284)	0.05(414)	1835	0.975(170)	0.047(248)	1101
	0.6	0.8	0.978(431)	0.048(605)	1185	0.979(142)	0.049(200)	395	0.976(84)	0.048(119)	237
	0.6	0.9	0.984(297)	0.045(375)	442	0.984(98)	0.047(124)	147	0.985(58)	0.046(74)	88
	0.7	0.8	0.974(846)	0.048(1222)	3930	0.973(281)	0.05(410)	1310	0.971(167)	0.049(245)	786
	0.7	0.9	0.979(431)	0.049(575)	789	0.978(142)	0.046(190)	263	0.976(84)	0.049(114)	158
	0.8	0.9	0.971(837)	0.048(1195)	2357	0.971(277)	0.051(398)	786	0.97(165)	0.049(238)	471
50%TPPR	0.5	0.6	0.802(3974)	0.05(4050)	7084	0.806(1321)	0.049(1347)	2361	0.8(792)	0.049(807)	1417
	0.5	0.7	0.82(1013)	0.048(1082)	1585	0.822(336)	0.52(358)	528	0.818(200)	0.05(214)	317
	0.5	0.8	0.856(462)	0.049(517)	622	0.854(153)	0.046(171)	207	0.854(91)	0.048(102)	124
	0.5	0.9	0.911(270)	0.043(298)	303	0.908(89)	0.046(98)	101	0.905(52)	0.047(58)	61
	0.6	0.7	0.809(3175)	0.049(3277)	5505	0.805(1056)	0.05(1090	1835	0.804(633)	0.049(653)	1101
	0.6	0.8	0.839(809)	0.051(891)	1185	0.838(268)	0.052(295)	395	0.835(160)	0.052(176)	237
	0.6	0.9	0.885(371)	0.046(416)	442	0.888(122)	0.047(137)	147	0.881(72)	0.047(82)	88
	0.7	0.8	0.809(2379)	0.053(2513)	3930	0.813(792)	0.052(836)	1310	0.812(474)	0.053(500)	786
	0.7	0.9	0.863(607)	0.05(687)	789	0.868(201)	0.052(228)	263	0.864(120)	0.051(136)	158
	0.8	0.9	0.832(1585)	0.05(1753)	2357	0.836(528)	0.051(583)	786	0.832(316)	0.051(349)	471
Maximal negative TPPR	0.5	0.6	0.796(7105)	0.05(7109)	7084	0.797(2360)	0.49(2361)	2361	0.797(1413)	0.05(1414)	1417
	0.5	0.7	0.812(1608)	0.047(1609)	1585	0.804(533)	0.051(534)	528	0.81(318)	0.05(318)	317
	0.5	0.8	0.827(639)	0.05(641)	622	0.829(211)	0.049(212)	207	0.832(126)	0.049(126)	124
	0.5	0.9	0.87(312)	0.05(317)	303	0.867(103)	0.048(104)	101	0.868(61)	0.048(62)	61
	0.6	0.7	0.798(5522)	0.05(5519)	5505	0.796(1836)	0.049(1836)	1835	0.798(1099)	0.05(1099)	1101
	0.6	0.8	0.812(1204)	0.051(1205)	1185	0.0818(399)	0.051(400)	395	0.812(238)	0.051(238)	237
	0.6	0.9	0.844(451)	0.048(456)	442	0.842(149)	0.047(151)	147	0.839(89)	0.047(90)	88
	0.7	0.8	0.799(3944)	0.049(3943)	3930	0.806(1311)	0.049(1311)	1310	0.803(785)	0.048(785)	786
	0.7	0.9	0.824(799)	0.052(803)	789	0.826(265)	0.05(266)	263	0.824(158)	0.05(159)	158
	0.8	0.9	0.806(2366)	0.052(2368)	2357	0.808(787)	0.05(788)	786	0.808(471)	0.051(471)	471

### Application revisited

Given the robustness of the proposed method of sample size recalculation described and validated in simulation above, we return to apply it to the application presented earlier in this paper. The cell probability values at maximum positive conditional dependence for diseased patients under the specified values of *TPR*_*A*_ and *TPR*_*B*_ are ${\widehat{p}}_1=0.81,{\widehat{p}}_2=0.09$, ${\widehat{p}}_3=0$, ${\widehat{p}}_4=0.1$. The cell probability values at maximum negative conditional dependence for diseased patients under the specified values of *TPR*_*A*_ and *TPR*_*B*_ are ${\widehat{p}}_1=0.71,{\widehat{p}}_2=0.19$, ${\widehat{p}}_3=0.10$, ${\widehat{p}}_4=0$. Table 7 shows an example range of the permissible values under the specified values of *TPR*_*A*_ and *TPR*_*B*_. Given this, we can create a likelihood of our observed interim data having come from each possible configuration of the alternative hypothesis using equation 3.

**Table 7:** Example range of cell probabilities based on: TPR_A_= 0.9 and TPR_B_ = 0.81

*p*_1_	*p*_2_	*p*_3_	*p*_4_	*TPR*_*A*_	*TPR*_*B*_
0.81	0.09	0.00	0.10	0.9	0.81
0.80	0.10	0.01	0.09	0.9	0.81
...	...	...	...	...	...
0.72	0.18	0.09	0.01	0.9	0.81
0.71	0.19	0.10	0.00	0.9	0.81

Applying the method outlined in section 3, we take; $\widehat{{\  TPR}_A}=0.9$, $\widehat{{\  TPR}_B}=0.81$, observed ${\widehat{n}}_A=66$, ${\widehat{n}}_B=3,\kern0.5em {\widehat{n}}_C=3$, ${\widehat{n}}_D=10$ and ${\widehat{n}}_E + {\widehat{n}}_F+{\widehat{n}}_G+\kern0.5em {\widehat{n}}_H=105$, implying $\widehat{\pi} = 0.439$. Using equation 4 the maximum likelihood value of $\widehat{TPPR}$ is 0.793. Given the fact that *π* is binomially distributed, the maximum likelihood estimate for the prevalence is equal to the observed prevalence, $\widehat{\pi\ }$. Taking these values and inserting them into equation 1 we get the value for the sample size required for sensitivity as **275**. Taking $\widehat{\ {TNR}_A}=0.8$ and $\widehat{TNR_B}=0.66$, with the observed values ${\widehat{n}}_E=21$, ${\widehat{n}}_F=4,\kern0.5em {\widehat{n}}_G=11$, ${\widehat{n}}_G=69$ and ${\widehat{n}}_A + {\widehat{n}}_B+{\widehat{n}}_C+\kern0.5em {\widehat{n}}_D=82$, implying $1-\widehat{\pi} = 0.561$. Using equation 3 to derive the maximum likelihood of the cell probabilities for specificity we estimate that $\widehat{TNNR}=0.635$. Inserting these values into equation 2 gives us a sample size estimate of **136.** Thus, the updated sample size, in order to use the interim information about the conditional dependence between the tests and to preserve a minimal nominal power of 0.8 should be **275**.

## Discussion

This paper has presented a robust method of sample size re-estimation for use in paired diagnostic accuracy studies where the conditional independence between the two tests may be unknown or inaccurately estimated at the start of the study. In terms of the recommendation of sample size estimation for the experiment as a whole a specific protocol is suggested given the results. Rather than basing the estimate for the experiment as a whole on the case where there is the maximal negative conditional dependence between tests – thus the largest possible sample size - as suggested in Alonzo et al. [21], we would suggest an alternative strategy, the robustness of which is highlighted in Table 6. Specifically, initially estimating the sample size at the maximal positive conditional dependence between tests, i.e. using *TPPR* = *TPR*_*B*_ - giving the smallest possible sample size - then, re-estimating the final sample size using the method simulated in Table 6. As long as the initial estimate for prevalence is close to accurate, this protocol is deemed appropriate as it balances the risk of collecting more participants than might actually be needed with collecting the most information about the true conditional dependence at the interim. Table 6 provides strong evidence for the integrity of this method in providing at minimum the nominal power while reducing the sample size when we have a higher than maximally negative true conditional dependence. Should the interim sample size be some other value, the maximum likelihood method will still be appropriate, although it should be kept in mind that the larger the interim sample size, as a proportion of the total possible sample size, the more accurate the interim sample size estimates will be, for individual cases.

Interestingly, the sample size values in the table seem to be somewhat greater, even when using our method than those typically seen in the literature in diagnostic test accuracy studies, see for example van Enst et al. [29] Although it is difficult to know the specifics of the 859 studies mentioned in the van Enst collection of meta-analyses, e.g. clinically significant differences, sample size estimation and hypothesis testing procedures, it is striking that the 50% covariance sample size is only 87 (IQR 45–185) participants. Very few of our sample sizes in Table 6 are this low for the size of effect (ratios) we are considering, even using our method of sample size reduction. It may be that many diagnostic accuracy studies commissioned do not carefully consider their sample sizes. While the method discussed here of estimating the conditional dependence between the tests via maximum likelihood, given constraints imposed by the specified marginals and under a multinomial model, is pertinent to paired diagnostic accuracy tests, there is little reason why similar processes could not be extended to similar problems. The kernel of the method, maximum likelihood estimation of the parameter related to the conditional dependence using a constrained multinomial model, is equally valid in other applications involving sample size re-estimation for paired binary 2 × 2 tables.

## Conclusions

In this paper we have described a sample size re-estimation procedure that can be applied in an interim analysis for a diagnostic test study that is comparing two methods of testing on patients that are being followed up over a period of time. The procedure uses information on the levels of conditional dependence between the two tests at the interim in order to refine the required sample size for a paired diagnostic accuracy study with a binary response. Evidence from simulations has been provided to demonstrate its functionality under various parameter values thought to reflect a range of commonly occurring situations. The procedure can be applied in the case of paired comparative diagnostic accuracy studies in order to more accurately gauge the sample size required for a given power thereby reducing both the costs associated with this kind of study and also the burden on patients.

## Appendix R code for maximum likelihood sample size re-estimation

ss.est.mle <- function(obs.a, obs.b, obs.c, obs.d, obs.x, tpra, tprb, alpha, beta){mle.tppr <- function(theta.1, obs.a, obs.b, obs.c, obs.d, tpra, tprb){-((obs.a*log(theta.1)) + obs.b*log(tpra-theta.1) + obs.c*log(tprb-theta.1) + obs.d*log(-tpra-tprb+theta.1+1))}tppr <- optim(par=(tpra+(tprb-(1-tpra)))/2 ,fn=mle.tppr, obs.a=obs.a, obs.b=obs.b, obs.c=obs.c, obs.d=obs.d, tpra=tpra, tprb=tprb, method = "Brent", lower=(tprb-(1-tpra)), upper=tprb)$parobs.prev <- (obs.a+obs.b+obs.c+obs.d)/(obs.a+obs.b+obs.c+obs.d+obs.x)alonzo <- function(lambda, prev ,beta, alpha, tprb, gam1){(((qnorm(1-beta) + qnorm(1-alpha))/log(gam1))^2 * (((gam1+1) * tprb)-(2 * lambda))/(gam1*tprb^2))/prev}gam1 <- tpra/tprbss.est <- alonzo(tppr, obs.prev, beta = beta, alpha = alpha, tprb = tprb, gam1 = gam1 )return(ss.est)}### Example sensitivityss.est.mle(obs.a=66, obs.b=3, obs.c=3, obs.d=10, obs.x=105, tpra=0.9, tprb=0.81, alpha=0.025, beta=0.2)### Example specificityss.est.mle(obs.a=69, obs.b=11, obs.c=4, obs.d=21, obs.x=82, tpra=0.8, tprb=0.66, alpha=0.025, beta=0.2)

## Abbreviations

CT: computed tomography
PET: Positron Emission Tomography
RMSE: Root Mean Squared Error
*TBR*_*A*_: True negative rate for test A
*TBR*_*B*_: True negative rate for test B
*TNNR*: True negative negative rate
*TPPR*: True positive positive rate
*TPR*_*A*_: True positive rate for test A
*TPR*_*B*_: True positive rate for test B

## References

[1] Knottnerus JA, van Weel C, Muris JWM (2002). Evaluation of diagnostic procedures. Br Med J.

[2] Swets JA, Pickett RM (1982). Evaluation of Diagnostic Systems.

[3] Zhou XH, Obuchowski NA, DK MC (2002). Statistical Methods in Diagnostic Medicine.

[4] Freedman LS (1987). Evaluating and comparing imaging techniques: a review and classification of study designs. Br J Radiol.

[5] Gerke O, Vach W, Høilund-Carlsen PF. PET/CT in Cancer - Methodological Considerations for Comparative Diagnostic Phase II Studies with Paired Binary Data. Methods Inf. Med. [Internet]. 2008 [cited 2014 Oct 15];470–9. Available from: http://www.schattauer.de/index.php?id=1214&doi=10.3414/ME0540. 19057803

[6] Gould AL (2001). Sample size re-estimation: recent developments and practical considerations. Stat Med.

[7] Wittes J, Brittain E (1990). The role of internal pilot studies in increasing the efficiency of clinical trials. Stat Med.

[8] Shih WJ, Peace K (1992). Sample size reestimation in clinical trials. Biopharm. Seq. Stat. Appl.

[9] Shih WJ (1993). Sample size reestimation for triple blind clinical trials. Drug Inf J.

[10] Gould AL, Shih WJ (1992). Sample size re-estimation without unblinding for normally distributed outcomes with unknown variance. Commun Stat Methods.

[11] Birkett MA, Day SJ (1994). Internal Pilot Studies for Estimating Sample Size. Stat Med.

[12] Gould AL (1992). Interim Analyses for Monitoring Clinical Trails that do not Affect Type I Error Rates. Stat Med.

[13] Herson J, Wittes J (1993). The Use of Interim Analysis for Sample Size Adjustment. Drug Inf J.

[14] Shih WJ, Zhao P (1997). Design for Sample Size Re-estimation with Interim Data for Double-Blind Clinical Trails. Stat Med.

[15] Proschan MA (2005). Two-stage sample size re-estimation based on a nuisance parameter: a review. J Biopharm Stat.

[16] Gerke O, Høilund-carlsen PF, Poulsen MH, Vach W (2012). Interim analyses in diagnostic versus treatment studies : differences and similarities. Am J Nucl Med Mol Imaging.

[17] Lijmer JG, Jeroen G (1999). Empirical Evidence of Design-Related Bias. JAMA.

[18] Lord SJ, Staub LP, Bossuyt PMM, Irwig LM (2011). Target practice : choosing target conditions for test accuracy studies that are relevant to clinical practice. Br Med J.

[19] Newcombe RG (1998). Improved Confidence Intervals for the Difference between Binomial Proportions Based on Paired Data. Stat Med.

[20] Tango T (1998). Equivalence Test and Confidence Interval for the Difference in the Proportions Based on Paired Data. Stat Med.

[21] Alonzo TA, Pepe MS, Moskowitz CS (2002). Sample Size Calculations for Comparative Studies of Medical Tests for Detecting Presence of Disease. Stat Med.

[22] Lu Y, Jin H, Genant HK (2006). On the Non-Inferiority of a Diagnostic Test Based on Paired Observations. Stat Med.

[23] Moskowitz CS, Pepe MS (2006). Comparing the Predictive Values of Diagnostic Tests: Sample Size and Analysis for Paired Study Designs. Clin Trials.

[24] Bonett DG, Price RM (2006). Confidence Intervals for a Ratio of Binomial Proportions Based on Paired Data. Stat Med.

[25] Vacek P (1985). The effect of conditional dependence on the evaluation of diagnostic tests. Biometrics.

[26] van Smeden M, Naaktgeboren CA, Reitsma JB, Moons KGM, de Groot JA (2014). Latent Class Models in Diagnostic Studies When There is No Reference Standard — A Systematic Review. Am J Epidemiol.

[27] Schiller I, van Smeden M, Hadgu A, Libman M, Reitsma B, Dendukuri N (2016). Bias due to composite reference standards in diagnostic accuracy studies. Stat Med.

[28] Royston P (1993). Exact conditional and unconditional sample size for pair-matched studies with binary outcome: a practical guide. Stat Med.

[29] van Enst WA, Naaktgeboren CA, Ochodo EA, de Groot JA, Leeflang MM, Reitsma JB (2015). Small-study effects and time trends in diagnostic test accuracy meta-analyses : a meta-epidemiological study. Syst Rev.

